# Acquired diaphragmatic hernia following a peritoneal biopsy for gastric cancer dissemination in the diaphragm: a case report

**DOI:** 10.1186/s40792-023-01685-w

**Published:** 2023-06-25

**Authors:** Kazuya Endo, Kentaro Hara, Koichi Nemoto, Nozomi Goto, Kazuhisa Nishina, Nozomi Funatsu, Maki Takagi, Kohdai Ueno, Atsushi Onodera, Haruhiko Cho

**Affiliations:** Department of Gastric Surgery, Tokyo Metropolitan Cancer and Infectious Diseases Center, Komagome Hospital, 3-18-22 Honkomagome, Bunkyo-Ku, Tokyo, 113-8677 Japan

**Keywords:** Diaphragmatic hernia, Staging laparoscopy, Laparoscopic repair

## Abstract

**Background:**

Acute diaphragmatic hernia is a life-threatening condition caused by prolapse of an abdominal organ into the thoracic cavity through a defect in the diaphragm. We present herein a case of acquired diaphragmatic hernia following a peritoneal biopsy for gastric cancer dissemination in the diaphragm.

**Case presentation:**

A 72-year-old, female patient presented with a complaint of acute abdomen 10 months after receiving a diagnosis of stage IV gastric cancer with peritoneal dissemination based on peritoneal biopsy findings during staging laparoscopy. Computed tomography demonstrated herniation of the small intestine into the thoracic cavity. Emergency surgery was performed, and a full-thickness diaphragmatic defect was found intraoperatively at the same location as the previous, peritoneal biopsy. The incarcerated small intestine was atraumatically repositioned into the abdominal cavity, and the defect was closed laparoscopically using an absorbable barbed suture.

**Conclusions:**

Although complications of staging laparoscopy are extremely rare, excising disseminated nodules from the diaphragm carries the risk of diaphragmatic hernia. For this reason, avoiding excision is desirable unless a diaphragmatic biopsy is needed.

## Background

Diaphragmatic hernia is classified into a congenital or acquired type, but most cases are congenital [1–6]. Acquired diaphragmatic hernia occurs after a trauma or as a complication of a medical procedure. Incarceration and strangulation of the bowel can be life-threatening, with the associated overall mortality rate being as high as 31% [1]. Moreover, acquired diaphragmatic hernia presents no typical, thoracic or abdominal symptoms and has an unexpected onset [7]. We present herein a case of acquired diaphragmatic hernia following a peritoneal biopsy for gastric cancer dissemination in the diaphragm which was repaired laparoscopically.

## Case presentation

**Fig. 1:**
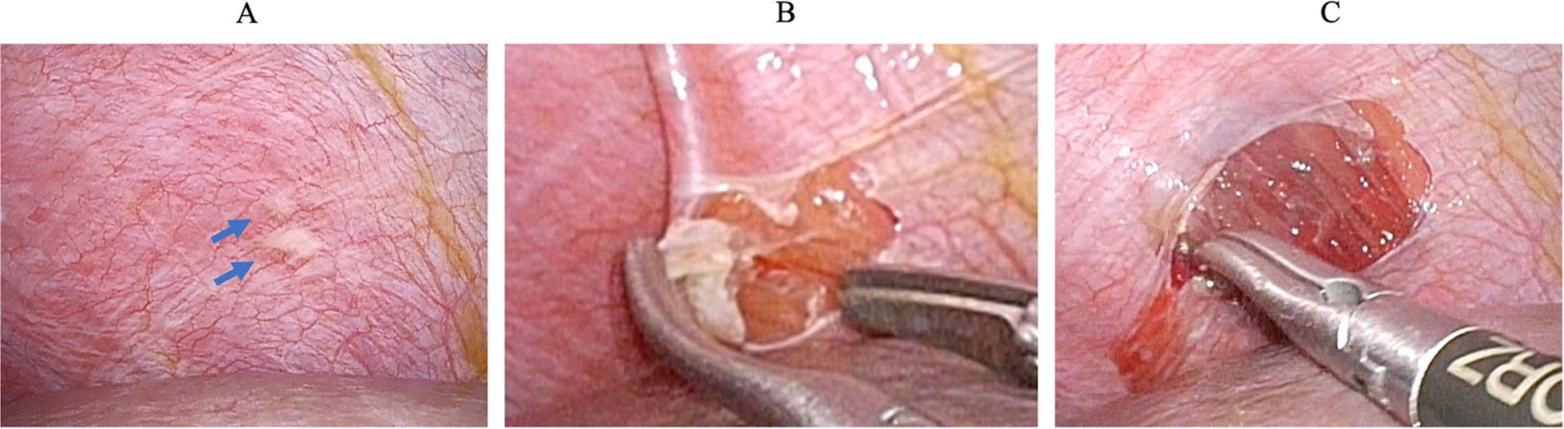
Images show the diaphragmatic peritoneal surface during the first surgery. **A, B** White nodules were observed on the diaphragmatic peritoneal surface. Analysis of an excision specimen led to the diagnosis of a peritoneal metastasis. **C** Hemostasis after nodule excision

A 72-year-old, female patient with advanced gastric cancer underwent staging laparoscopy for suspected peritoneal dissemination 10 months previously. The laparoscopic examination found a small nodule in the peritoneum of the right diaphragm, which was excised for analysis using laparoscopic scissors. The peritoneal defect caused by the biopsy did not penetrate the diaphragm and was small enough (about 2 cm in diameter) not to require suturing or reinforcement (Fig. 1A, B). The slight bleeding that occurred after the excisional biopsy was successfully stopped using a soft-coagulation system (Valleylab™ FT10 SOFT COAG mode 60W, Fig. 1C). Microscopically the nodule was found to be a disseminated adenocarcinoma. Gastric adenocarcinoma cT4a(SE)N0M1(PER) cStageIVB was diagnosed based on the UICC–TNM classification, 8th edition.

**Fig. 2:**
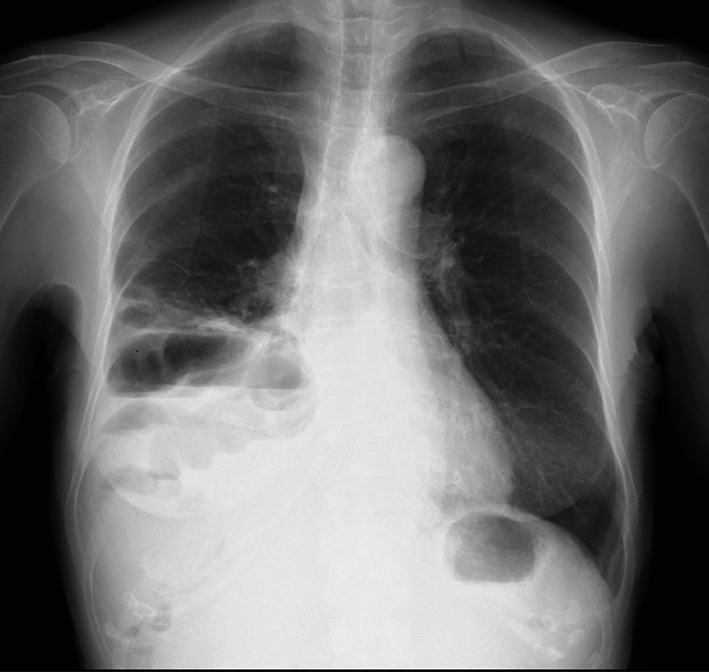
Chest X-ray

**Fig. 3:**
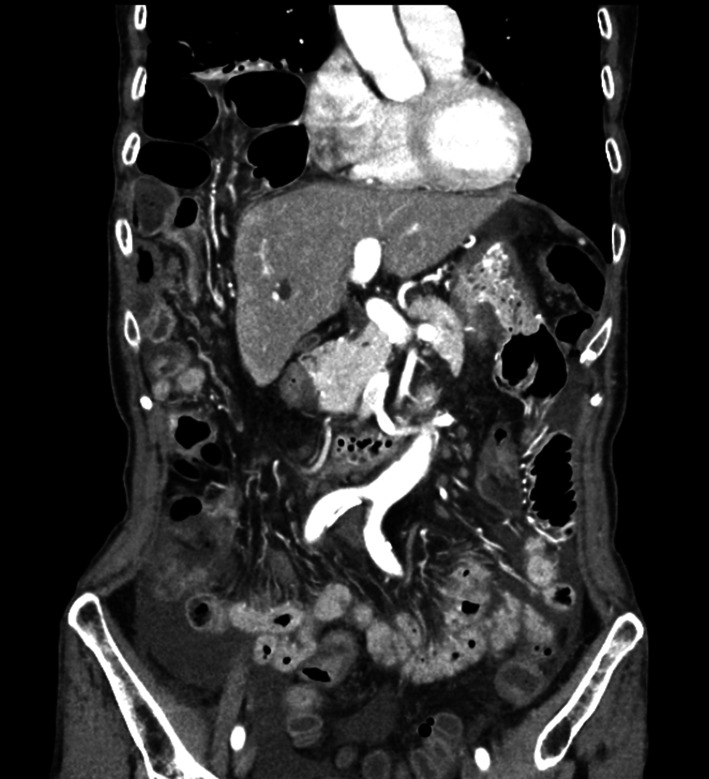
Contrast-enhanced computed tomography demonstrating herniation of the small intestine into the thoracic cavity

The patient received S1, oxaliplatin, and nivolumab as first-line chemotherapy. After 11 courses of chemotherapy, she experienced a sudden onset of epigastric pain and visited the emergency department. She had mild tenderness around the upper abdomen without any signs of peritonitis. She had no history of trauma. Laboratory tests found no specific abnormalities. A chest X-ray (Fig. 2) and contrast-enhanced computed tomography (CT) (Fig. 3) revealed prolapse of the small intestine into the thoracic cavity without ischemic changes. Based on these findings, acute diaphragmatic hernia without strangulation was diagnosed.

**Fig. 4:**
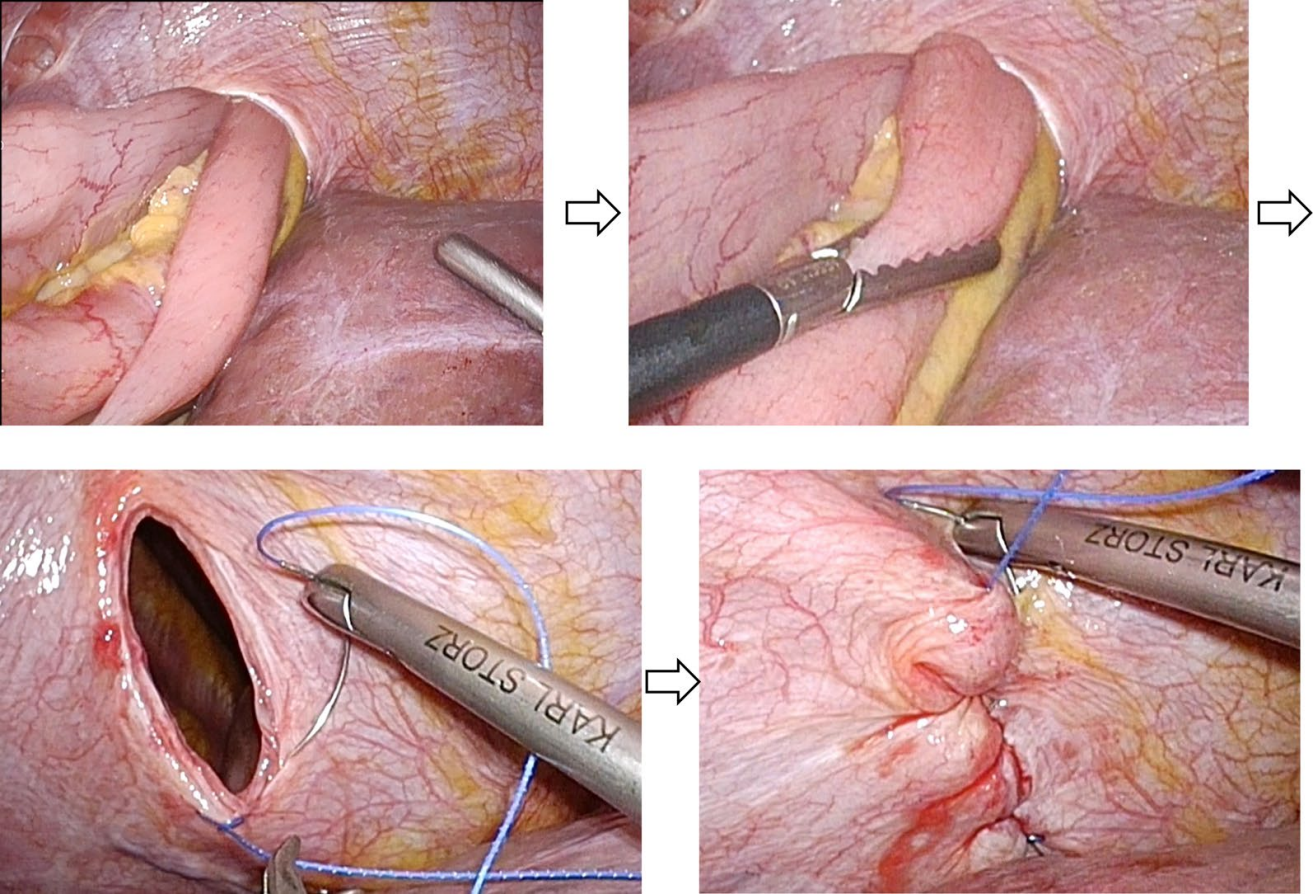
Herniated small intestine, repositioning, and closure of the hernial orifice

Emergency laparoscopy was performed. Intraoperatively, the small intestine was found to have herniated into the right thoracic cavity through a diaphragmatic defect corresponding to the site of the previous peritoneal biopsy. The peritoneal disseminated nodules had resolved, and there was no sign of a recurrence. The incarcerated small intestine was atraumatically repositioned into the abdominal cavity. As there were no ischemic changes, no resection was required. The diameter of the hernial orifice had expanded to approximately 4 cm and was closed using a non-absorbable, 3–0 barbed suture (Fig. 4). The operative time was 70 min, and blood loss was minimal. The postoperative course was uneventful.

## Discussion

We presented a case of acquired diaphragmatic hernia following a peritoneal biopsy for gastric cancer dissemination in the diaphragm which was successfully repaired laparoscopically. The present case highlights the risk of diaphragmatic hernia as a complication of diaphragmatic peritoneal biopsies.

Acquired diaphragmatic hernia occurs after trauma or as a complication of a medical procedure. Although an excisional biopsy of peritoneal lesions on the diaphragmatic surface is often performed during diagnostic laparoscopy, diaphragmatic hernias are not a common, late complication of the procedure, and only two cases, including the present one, have thus far been reported [8]. As the diaphragm in the present case had not been penetrated while performing the peritoneal surface biopsy, the delayed perforation was thought to have occurred as a result of an increase in the pleuroperitoneal pressure gradient, which can rise to + 100 cmH_2_O with maximal inspiration. No residual tumors were observed around the excised diaphragm; therefore, the perforation of the diaphragm was considered to be unassociated with tumor necrosis caused by the chemotherapy.

Another concern was the coagulation procedure used for hemostasis after peritoneal resection during the staging laparoscopy. Previous reports of diaphragmatic hernia associated with a medical procedure also speculated that the diaphragm may be inadvertently injured intraoperatively through contact with energy devices, such as ultrasonic coagulation shears, or during electrocautery [8–10]. The findings in the present case indicate the importance of avoiding the diaphragm when performing a peritoneal biopsy and of avoiding heat coagulation for hemostasis; soft-coagulation mode electricity for hemostasis was used in this case. Whenever possible, it is important to avoid performing a biopsy of the diaphragm. In case where it is unavoidable, it may be necessary to forego coagulation during bleeding and instead consider using sutures for hemostasis and reinforcement, regardless of the size or depth of the defect.

Diaphragmatic hernia is diagnosed on the basis of clinical presentations and confirmed by imaging studies, such as X-ray, contrast-enhanced CT, and magnetic resonance imaging. Common, radiological findings include an elevated hemidiaphragm, blunting of the costophrenic angle, distortion of the diaphragm borders, curling of the gastric tube into the thorax, mediastinal shift, and pleural effusion or presence of air-filled gastrointestinal structures in the thoracic cavity [2, 3]. CT has a sensitivity of 55% and specificity of 100% for diagnosing diaphragmatic hernia [6]. In the present case, a chest X-ray revealed a right elevation of the diaphragm and the presence of the small bowel in the right thoracic cavity, while contrast-enhanced CT demonstrated herniation of the small intestine into the thoracic cavity.

Acquired diaphragmatic hernia is fatal unless emergency surgery is performed to prevent bowel obstruction, ischemia, and perforation, any which can have life-threatening consequences [7]. The optimal treatment for this condition has not been established. However, laparoscopy may be safer and more feasible than open surgery, considering the minimal impact it has on the abdominal wall and recovery of bowel movement. Furthermore, laparoscopy is useful for securing a wider, surgical field-of-view, as the defect is located deeply in the cranial side of the abdominal cavity [4, 5]. In the present case, laparoscopy provided excellent visualization and easy access to the diaphragmatic hernial orifice, so that a smaller incision was able to be made than required by open surgery.

Mesh reinforcement of the repair site depends on the defect size (> 8 cm) or the fragility of the diaphragmatic tissue around the hernial defect [2]. In the present case, the peritoneal defect was 4 cm in diameter and was closed using a non-absorbable, 3–0 barbed suture.

## Conclusions

We reported a case of acquired diaphragmatic hernia which occurred after laparoscopic excision of a diaphragmatic surface nodule. Although complications of staging laparoscopy are extremely rare, excising disseminated nodules from the diaphragm carries the risk of diaphragmatic hernia. For this reason, avoiding excision is desirable unless a diaphragmatic biopsy is needed.

## Data Availability

The data in this study are available from the corresponding author upon reasonable request.

## Abbreviation

CT: Computed tomography
